# Localization of Hippo signalling complexes and Warts activation *in vivo*

**DOI:** 10.1038/ncomms9402

**Published:** 2015-09-30

**Authors:** Shuguo Sun, B. V. V. G. Reddy, Kenneth D. Irvine

**Affiliations:** 1 Howard Hughes Medical Institute, Waksman Institute and Department of Molecular Biology and Biochemistry, Rutgers University, Piscataway, New Jersey 08854, USA

## Abstract

Hippo signalling controls organ growth and cell fate by regulating the activity of the kinase Warts. Multiple Hippo pathway components localize to apical junctions in epithelial cells, but the spatial and functional relationships among components have not been clarified, nor is it known where Warts activation occurs. We report here that Hippo pathway components in *Drosophila* wing imaginal discs are organized into distinct junctional complexes, including separate distributions for Salvador, Expanded, Warts and Hippo. These complexes are reorganized on Hippo pathway activation, when Warts shifts from associating with its inhibitor Jub to its activator Expanded, and Hippo concentrates at Salvador sites. We identify mechanisms promoting Warts relocalization, and using a phospho-specific antisera and genetic manipulations, identify where Warts activation occurs: at apical junctions where Expanded, Salvador, Hippo and Warts overlap. Our observations define spatial relationships among Hippo signalling components and establish the functional importance of their localization to Warts activation.

## Abstract (web summary)

Components of the Hippo signalling pathway localize to apical junctions in epithelial cells, where they regulate growth in response to mechanical and biochemical cues. Sun *et al*. show that these proteins are organized into distinct junctional complexes, which reorganize up on Hippo pathway activation.

## Graphical abstract

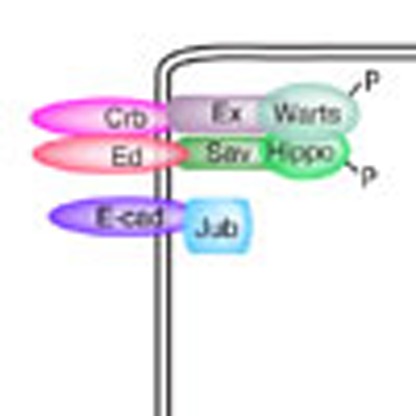

Hippo signalling is a conserved signal transduction pathway that controls organ growth and cell fate throughout the metazoa^1^,^2^,^3^. Hippo signalling is regulated by diverse inputs and subject to extensive crosstalk by other pathways, but major factors regulating the pathway include cellular organization and contacts with neighbouring cells. Thus, disruption of apical–basal polarity in epithelial cells inactivates Hippo signalling^4^, whereas contact inhibition is associated with high levels of Hippo signalling^5^. Genetic and biochemical studies have identified many components of Hippo signalling and characterized their potential for interactions, but our understanding of the normal cellular organization and dynamics of the Hippo pathway *in vivo* has remained relatively poor.

Hippo signalling regulates growth by inhibiting a transcriptional co-activator protein, Yorkie (Yki)^6^. Yki and its mammalian homologues are crucial and potent promoters of growth, consequently disruption of Hippo signalling is associated with many cancers^7^, whereas excess Hippo pathway activity inhibits growth and induces apoptosis. The key direct regulator of Yki activity is the kinase Warts (Wts): phosphorylation of Yki by Wts inactivates Yki by restricting it to the cytoplasm^8^,^9^. A complex network of regulatory proteins converge to modulate Wts activity. Direct activators of Wts include Hippo (Hpo), a kinase that activates Wts, Salvador (Sav), a scaffolding protein that binds both Hpo and Wts, and Mob as tumour suppressor (Mats), a Wts co-factor (Fig. 1a)^1^,^3^. Wts activation is promoted by cytoplasmic proteins associated with apical junctions, including Kibra, Merlin (Mer) and Expanded (Ex)^10^,^11^,^12^,^13^,^14^. Activated forms of Mer can bind to Wts, and it has been proposed that Mer activates Wts by recruiting it to membranes where it could be phosphorylated by Hpo^15^. However, inhibitors of Wts activity also localize to junctional membrane complexes, including Zyxin^16^,^17^, and Ajuba LIM protein (Jub)^18^,^19^. Relationships among the various junctional components of Hippo signalling have not been well defined, and where within the cell Wts activation occurs has not been determined.

We report here that Hippo pathway components in the *Drosophila* wing disc are organized into distinct complexes at apical junctions, including separate distributions for Sav, Ex, Wts and Hpo. These complexes are reorganized on Hippo pathway activation, when Wts shifts from associating with the Wts inhibitor Jub to the Wts activator Ex, and Hpo shifts from predominantly cytoplasmic to predominantly associating with Sav. We identify both increased expression of Wts and Ex, and increased Hpo activity, as contributing to this Warts relocalization. Using a phospho-specific antisera, we find that Wts is activated at sub-apical junctions where Ex, Sav, Hpo and Wts overlap. We also define the role of Ex within the Hippo pathway as functioning as a scaffold that recruits Wts to membrane locations where it interacts with and can be activated by Hpo. Our observations identify sites of Wts activation *in vivo*, define spatial relationships among Hippo signalling components and establish the functional importance of their localization to Wts activation.

## Results

### Organization of Hippo signalling components in wild type

Physical interactions among Hippo pathway components have been characterized mostly based on overexpression in cultured cells. However, in many cases, the extent to which such interactions occur *in vivo* remains unclear, as regulated localization to separate sites within the cell could prohibit interactions, whereas localization to shared sites could promote them. To assess the potential for regulated localization to modulate Hippo signalling *in vivo*, we compared the subcellular distributions of Hippo pathway components expressed at endogenous levels using antibodies or tagged genomic constructs for Crumbs (Crb), Ex, Wts, Echinoid (Ed), Sav, Hpo, Mats and Jub in wing disc epithelial cells, and then examined how their localization was affected by pathway activation.

**Figure 1:**
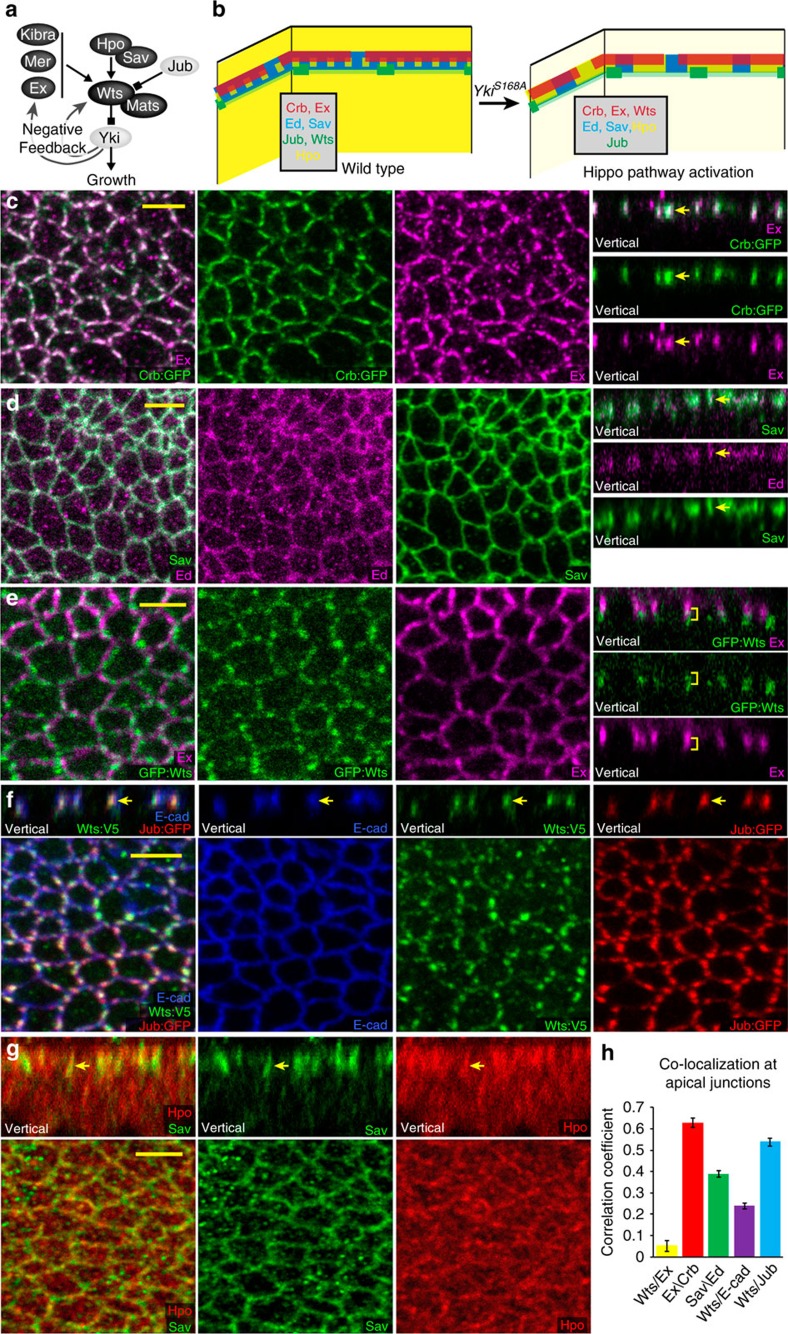
Apical organization of Hippo pathway components. (**a**) Schematic illustrating regulatory relationships among key Hippo pathway components, darkly shaded proteins are all inhibitors of Yki activity. (**b**) Cartoon illustrating relative location of Hippo pathway components at apical junctions in wild type, and in cells expressing Yki^S168A^. (**c**–**g**) Wing disc cells, including combined and individual stains as indicated, in horizontal and vertical (as marked) sections. Arrows highlight examples of overlapping puncta, yellow bracket highlights puncta at distinct apical–basal positions; scale bar (yellow), 3 μm. Panels show expression of: (**c**) Crb:GFP (green) and Ex (magenta), (**d**) Ed (magenta) and Sav (green), and (**e**) GFP:Wts (green) and Ex (magenta); the mean distance between puncta was 0.8 μm (*N*=7). (**f**) Jub:GFP (red), Wts:V5 (green) and E-cad (blue), (**g**) Sav (green) and Hpo (red). (**h**) Histogram showing Pearson's correlation coefficient for co-localization of the indicated pairs of protein stains in wild-type discs, calculated from five discs per comparison, error bars indicate s.d.

Kibra, Mer and Ex interact genetically and exhibit overlapping apical distributions^10^,^11^,^12^,^13^,^14^; we focused on Ex, as it is a major Hippo pathway activator in the wing disc, whereas Kibra and Mer have weaker effects. Ex localizes to apical junctions largely through association with the transmembrane protein Crb, which participates in homophilic interactions between cells; Crb and Ex exhibit extensive co-localization (Fig. 1c)^20^,^21^,^22^. Along the apical–basal axis, these proteins accumulate at a site known as the sub-apical region or marginal zone, which is just apical to the E-cadherin-containing adherens junctions (Supplementary Fig. 1a)^23^. Within the plane of the sub-apical region, Crb and Ex localization are discontinuous, with small gaps in their membrane accumulation around each cell (Fig. 1c; Supplementary Fig. 1a). Sav localizes to apical junctions largely through association with the transmembrane protein Ed, which also participates in homophilic interactions between cells, and Ed and Sav co-localize in wing discs (Fig. 1d)^24^. Ed–Sav complexes overlap Crb–Ex complexes (Supplementary Fig. 1a), but have a broader distribution within the horizontal plane, as there are no obvious gaps in their distribution (Fig. 1d), and they also partially overlap the more basal adherens junction complexes (Supplementary Fig. 1a). Wts co-localizes with the Wts inhibitor Jub (Fig. 1f), which is responsible for recruiting Wts to adherens junctions^18^. In late third instar wing discs, Jub–Wts complexes were ∼0.8 μm basal to Crb–Ex complexes (Fig. 1e). Wts and Jub exhibit a punctate distribution within the plane of the adherens junctions, which is distinct from the Crb–Ex distribution (Fig. 1e). Finally, Hpo localization is predominantly cytoplasmic, but appears slightly elevated at apical junctions^25^, in a punctate distribution, partially overlapping Sav (Fig. 1g; Supplementary Fig. 1b,c). The co-localization of Ex with Crb, Sav with Ed, and Wts with Jub was confirmed by calculation of Pearson's correlation coefficient for each of these pairs of interacting proteins at apical junctions (Fig. 1h). Altogether, this characterization identified four distinct localization profiles for Hippo pathway components: Crb–Ex, Ed–Sav and Jub–Wts complexes at distinct apical junctions, and a distribution of Hpo that includes both broad cytoplasmic accumulation and a modest junctional accumulation overlapping Sav (Fig. 1b).

### Detection of Wts activation *in vivo*

**Figure 2:**
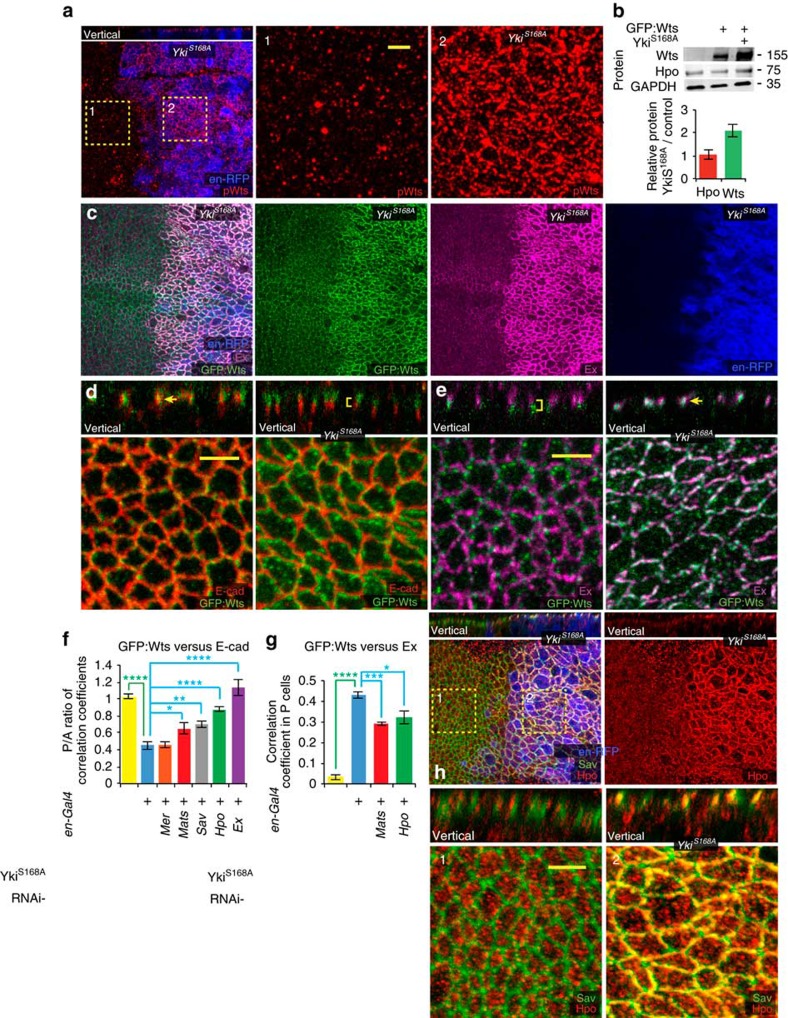
Relocalization of Wts and Hpo associated with Hippo pathway activation. (**a**) Wing disc with Yki^S168A^ expressed in posterior cells for 24 h under *en-Gal4* control, marked by *UAS-RFP* (blue), and stained for pWts (red). Panels on right marked by numbers show higher magnification of the boxed regions (−1 for anterior cells, −2 for posterior cells); scale bar (yellow), 3 μm. (**b**) Representive western blots on the indicated proteins (molecular weight on right) and results of quantification of four independent blots performed on wing discs lysates from wild type, *GFP:Wts* and *GFP:Wts en-Gal4 UAS-yki:V5*^*S168A*^ *tub-Gal80*^*ts*^ shifted to 29 °C 24 h; amounts were quantified and normalized to GAPDH. (**c**–**e**) Wing disc with Yki^S168A^ expressed in posterior cells for 24 h under *en-Gal4* control, including combined and individual stains as indicated, in horizontal and vertical (as marked) sections, marked by *UAS-RFP* (blue), and showing (**c**) GFP:Wts (green) and Ex (magenta), (**d**) GFP:Wts (green) and E-cad (red), (**e**) GFP:Wts (green) and Ex (magenta); for **d** and **e**, left-side panels show anterior (control) cells and right-side panels show posterior (Yki^S168A^-expressing) cells from the same disc, arrows highlight examples of overlapping puncta and yellow brackets highlight puncta at distinct apical–basal positions. (**f**) Histogram showing ratio between posterior and anterior cells of Pearson's correlation coefficient for co-localization of GFP:Wts and E-cad in discs expressing *en-Gal4 tub-Gal80*^*ts*^ (shifted to 29 °C for 24 h) and where indicated, *UAS-Yki:V5*^*S168A*^ and UAS-RNAi lines, based on five discs each. (**g**) Histogram showing Pearson's correlation coefficient for co-localization of GFP:Wts and Ex in posterior cells of discs expressing *en-Gal4 tub-Gal80*^*ts*^ (shifted to 29 °C for 24 h) and where indicated, *UAS-Yki:V5*^*S168A*^ and UAS-RNAi lines, based on five discs each. P/A ratios are not shown here because the correlation coefficient in A cells is close to 0. (**f**,**g**) Error bars indicate s.d., and asterisks indicate statistical significance of difference between selected pairs of results, determined by one-way analysis of variance. (**h**) Wing disc with Yki^S168A^ expressed in posterior cells for 24 h under *en-Gal4* control, marked by *UAS-RFP* (blue), and stained for Hpo (red) and Sav (green). Panels marked by numbers show higher magnification of the boxed regions (−1 for anterior cells, −2 for posterior cells); scale bar (yellow), 3 μm.

Although the cellular location of Wts activation has not been identified, activation of Wts is associated with Hpo-mediated phosphorylation at Thr1077, and an antisera that recognizes phospho-Thr1077 (pWts) on western blots has been reported^10^. Staining wild-type wing disc cells with this antisera revealed only nonspecific background staining, comprising both dispersed puncta and a Wts-independent mitotic stain (Supplementary Fig. 2a). However, the observation that Wts co-localizes with an inhibitor, Jub, rather than with the activators Ex, Sav or Hpo, implies that most Wts protein *in vivo* is inactive. Thus, we wondered whether it might be possible to visualize pWts *in vivo* under conditions of elevated Hippo pathway activity. Hippo pathway activation inhibits Yki, thereby decreasing growth and increasing apoptosis, whereas Yki activation promotes growth and blocks apoptosis^1^,^2^,^3^. Yki also enhances expression of several upstream Hippo pathway components (Fig. 1a)^11^,^13^, including Ex, Mer and Kibra, and as we show here, Wts (Fig. 2b,c; Supplementary Fig. 2b). We hypothesized that by expressing an activated form of Yki, these negative feedback loops could be used to activate Hippo signalling upstream of Yki (Fig. 1a), while simultaneously blocking downstream apoptosis. Thus, we expressed Yki containing a Ser-to-Ala mutation at the main Wts phosphorylation site (Yki^S168A^), which renders it constitutively activated and insensitive to upstream Hippo pathway activation^8^,^9^. Because of lethality associated with high-level activation of Yki, Yki^S168A^ was expressed under UAS-Gal4 control in a conditional manner, by combining *en-Gal4*, which drives expression of UAS transgenes in posterior cells, with *tub-Gal80*^*ts*^, which expresses a temperature-sensitive form of the Gal4 repressor Gal80. Examination of wing discs in which Yki^S168A^ was expressed in posterior cells of wing discs revealed a clear, Wts-dependent, pWts signal above the background staining (Fig. 2a; Supplementary Fig. 2c) Thus, under these conditions of Hippo pathway activation, activated Wts can be detected *in vivo*.

### Relocalization of Wts and Hpo induced by pathway activation

Since Wts normally co-localizes with an inhibitor rather than its activators, we wondered whether activation of Wts is associated with changes in the localization of pathway components. Remarkably, under conditions of Wts activation induced by activated Yki, Wts shifts from overlapping with E-cadherin (E-cad) at adherens junctions to overlapping with Ex at Crb–Ex junctions (Fig. 2d,e). Expression of wild-type Yki, which does not increase Yki activity^26^, did not affect Wts localization (Supplementary Fig. 2d). The shift in Wts localization induced by activated Yki was evident both in the more apical localization of Wts and in a more even distribution around the circumference of the cell, matching Ex, and was quantified by calculating Pearson's correlation coefficient, which confirmed both increased Ex–Wts co-localization and decreased E-cad–Wts co-localization (Fig. 2f,g). Total levels of Wts protein, as assayed by western blotting, were also increased by Yki^S168A^ (Fig. 2b), and this affect appears to be at least partially transcriptional because expression of ß-galactosidase from a *wts-lacZ* enhancer trap was also increased by Yki^S168A^ (Supplementary Fig. 2b). A similar shift in Wts localization, from overlapping E-cad to a more apical location, could also be induced by Yki^S168A^ expression in eye imaginal discs (Supplementary Fig. 3a).

Examination of additional Hippo pathway components revealed that Hpo localization is also altered in Yki^S168A^-expressing cells. Apical junction accumulation of Hpo was greatly increased (Fig. 2h), whereas total Hpo levels were not affected (Fig. 2b), indicating that Hpo shifts from a cytoplasmic towards an apical junction localization. The increased accumulation of Hpo at apical junctions overlapped Sav (Fig. 2h), but was less extensive, as some gaps in the Hpo distribution relative to Sav were observed (Fig. 2h). Thus, Wts and Hpo, the two key kinases of the Hippo signalling pathway, are both relocalized concomitant with Hippo pathway activation induced by Yki^S168A^ (Fig. 1b).

**Figure 3:**
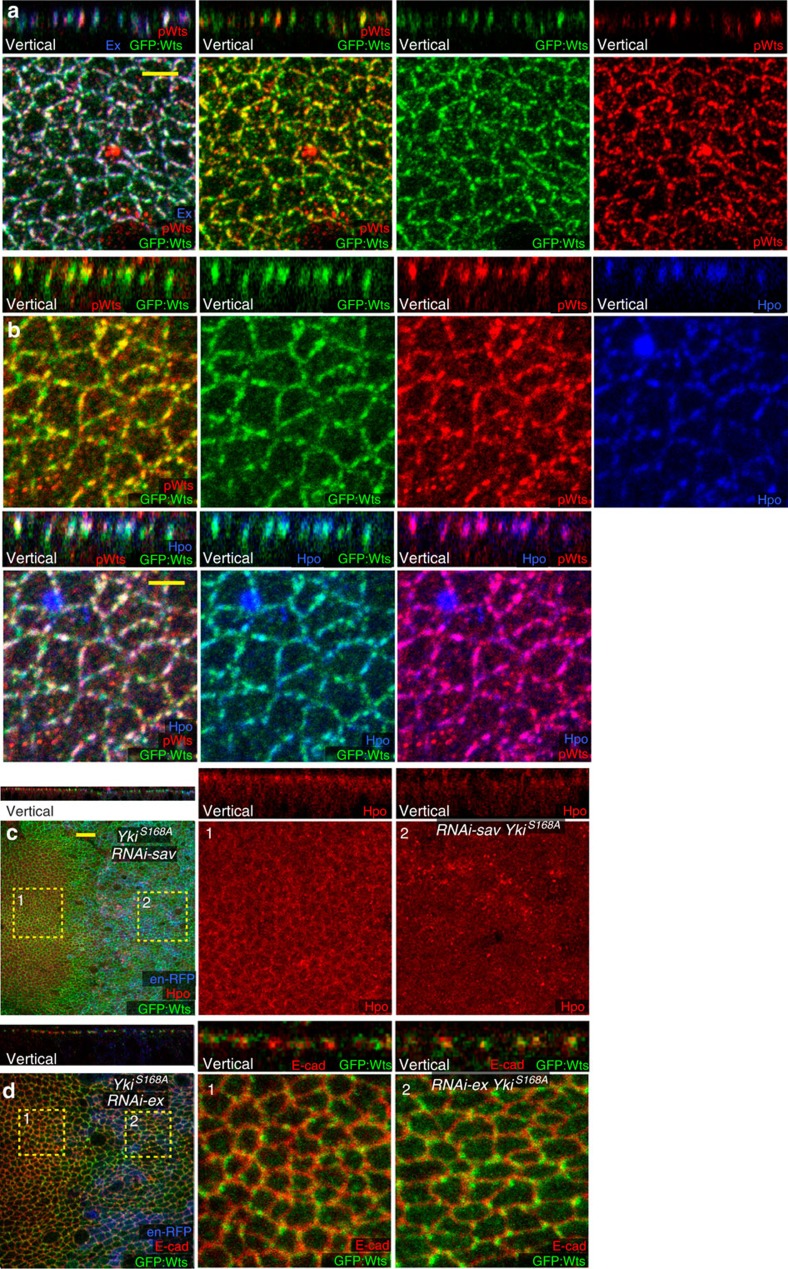
Localization of Wts activation *in vivo*. (**a**,**b**) Posterior wing disc cells expressing Yki^S168A^ for 24 h under *en-Gal4* control, including combined and individual stains as indicated, in horizontal and vertical (as marked) sections; scale bar (yellow), 3 μm. Panels show expression of: (**a**) Ex (blue), pWts (red), GFP:Wts (green), (**b**) Hpo (blue), pWts (red) and GFP:Wts (green). (**c**,**d**) Wing disc cells with Yki^S168A^ and *RNAi-sa*v (**c**) or *RNAi-ex* (**d**) expressed in posterior cells for 24 h under *en-Gal4* control, marked by *UAS-RFP* (blue), including combined and individual stains as indicated, in horizontal and vertical (as marked) sections, panels marked by numbers show higher magnification of the boxed regions (−1 for anterior cells, −2 for posterior cells); scale bar (yellow), 3 μm. Panels show expression of: (**c**) Hpo (red), GFP:Wts (green), (**d**) E-cad (red) and GFP:Wts (green).

To clarify the relationship between Hpo and Wts relocalization and Wts phosphorylation, we compared their distribution in cells expressing Yki^S168A^. pWts staining overlaps with Wts and Ex, although occasional sites of Wts accumulation without detectable pWts could be identified (Fig. 3a,b). pWts staining is also encompassed within apical Hpo accumulation (Fig. 3b), and simultaneous examination of pWts, Wts and Hpo revealed that pWts is detected at apical junctions where Hpo and Wts overlap (Fig. 3b). Thus, Wts is activated *in vivo* when and where the subcellular localizations of Hpo and Wts intersect. These results support a model in which pathway activation is controlled by regulating the subcellular localization of Wts and Hpo. We note that key accessory proteins, including Sav and Ex, also co-localize at these sites where pWts is detected, and could play an essential role in assembling a Wts activation complex.

### Mats–Wts association is independent of Wts activation

**Figure 4:**
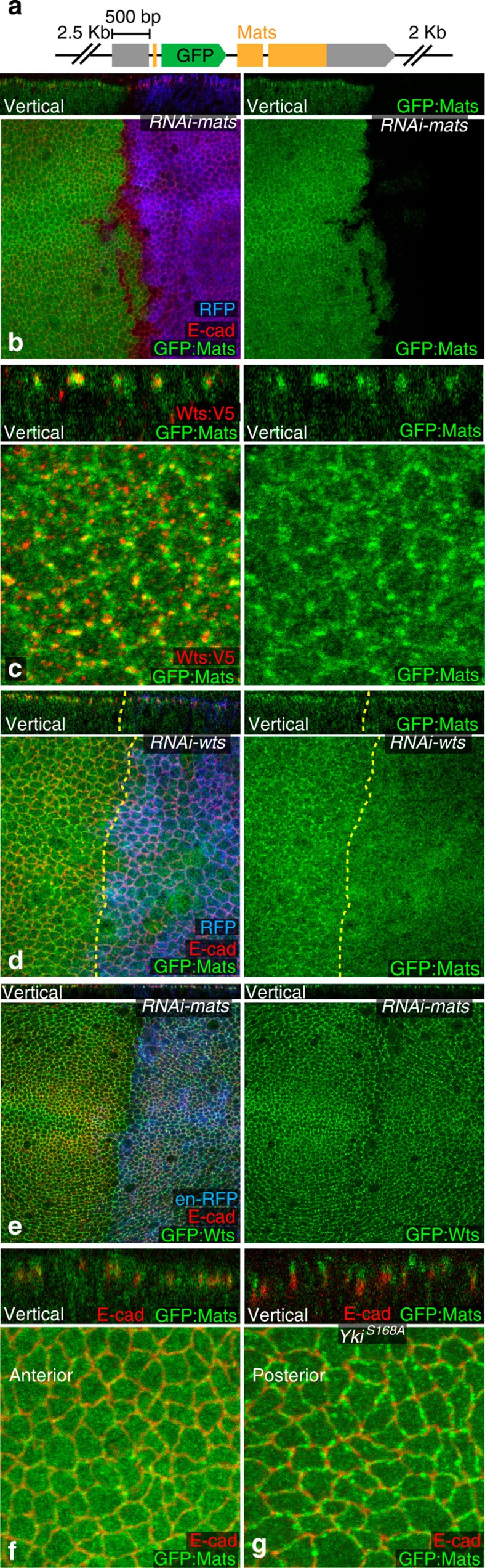
Co-localization of Mats with Wts. (**a**) Schematic of the genomic GFP:Mats construct, which includes 3-kb upstream and 3-kb downstream of the GFP insertion. Orange and grey boxes represent coding exons and untranslated regions, respectively. (**b**) GFP:Mats (green) expressing wing disc with *mats* RNAi expressed in posterior cells under *en-Gal4* control, marked by *UAS-RFP* (blue). (**c**) Wing disc cells expressing Wts:V5 (red) and GFP:Mats (green). (**d**) GFP:Mats (green) expressing wing disc with *wts* RNAi expressed in posterior cells under *en-Gal4* control, marked by *UAS-RFP* (blue), and stained for E-cad (red). Dashed yellow lines mark the A–P boundary. (**e**) GFP:Wts (green) expressing wing disc with *mats* RNAi expressed in posterior cells under *en-Gal4* control, marked by *UAS-RFP* (blue), and stained for E-cad (red). (**f**,**g**) GFP:Mats (green) expressing wing disc with Yki^S168A^ expressed in posterior cells under *en-Gal4* control, and stained for E-cad (red), close ups of anterior (control) cells (**f**), and posterior (Yki^S168A^-expressing) cells (**g**) from the same wing disc are shown. Panels including combined and individual stains as indicated, in horizontal and vertical (as marked) sections.

The Wts co-factor Mats has been reported to localize to both the cytoplasm and adherens junctions^27^. It was also reported that forced membrane localization of Mats, achieved by expressing a myristoylated Mats construct, Myr:Mats:green fluorescent protein (GFP), could recruit Wts to membranes and increase Wts activation^27^. Thus, we investigated the relationship between Wts relocalization and activation, and Mats localization. As available Mats antisera did not yield specific staining in our hands, we created a genomic GFP:Mats transgene (Fig. 4a). The specificity of GFP:Mats expression was confirmed by its depletion using *mats* RNA interference (RNAi; Fig. 4b). In addition to a broad cytoplasmic distribution, GFP:Mats localizes to discrete puncta at adherens junctions, which overlap Wts puncta (Fig. 4c). Thus, Mats co-localizes with Wts even in its inactive (Jub-associated) state. Mats is recruited to adherens junctions by Wts, because GFP:Mats localization to junctional puncta was eliminated by *wts* RNAi (Fig. 4d). Conversely, Wts localization to adherens junctions does not require Mats (Fig. 4e). Relocalization of Wts induced by Yki^S168A^ induced a concomitant relocalization of GFP:Mats to more apical junctions (Fig. 4f,g). Thus, we infer that *in vivo* Mats can associate with Wts at both inactive (Jub) and active (Ex) sites.

### Assembling a Wts activation complex

The implication that Wts activation could be ascribed to a relocalization of Wts and Hpo that facilitates their interaction prompted us to examine the requirements for Hpo pathway components in this process. Since the Hpo that accumulates at the apical membrane overlaps with Sav, we used RNAi to knockdown *sav* in cells expressing Yki^S168A^. This severely diminished apical accumulation of Hpo in Yki^S168A^-expressing cells, indicating that this recruitment of Hpo is Sav dependent (Fig. 3c). This observation is consistent with cell fractionation experiments in cultured cells reporting an ability of Sav to promote membrane association of Hpo^15^. The modest apical accumulation of Hpo in wild-type cells was also Sav dependent (Supplementary Fig. 3d). Thus, while most Hpo is normally cytoplasmic, Sav can recruit Hpo to apical junctions, and this recruitment can be enhanced by conditions that promote Hippo pathway activity.

Wts overlaps with Jub in wild-type but not in Yki^S168A^-expressing cells, and Jub is not required for the relocalization of Wts (Supplementary Fig. 3b,c). Conversely, relocalized Wts in Yki^S168A^-expressing cells overlaps extensively with Ex (Fig. 2e,g), and Ex was required for Wts relocalization: in cells co-expressing Yki^S168A^ and *ex*-RNAi, Wts remained at adherens junctions rather than shifting to Crb–Ex junctions (Fig. 3d; Fig. 2f). Knockdown of Ex also eliminated detectable pWts staining in Yki^S168A^-expressing cells (Supplementary Fig. 3e). Thus, Ex is required for both the relocalization and the activation of Wts induced by Yki^S168A^ in wing disc cells. Conversely, knockdown of Mer did not detectably influence Wts relocalization (Fig. 2f; Supplementary Figs 1d and 4a), consistent with Mer playing only a minor role in Wts activation in wing discs.

We also investigated requirements for Hpo, Mats and Sav in Wts relocalization. When Hpo, Mats or Sav levels were reduced by RNAi in Yki^S168A^-expressing cells, Wts relocalization was reduced, as revealed by its partial co-localization with both Ex and E-cad (Fig. 2f,g; Supplementary Fig. 4b–f). We also confirmed that Wts phosphorylation was severely diminished by *sav* RNAi, consistent with its role in localizing Hpo to apical junctions where Wts phosphorylation occurs (Supplementary Fig. 3f). These results indicate that Hpo, Sav and Mats each contribute to the relocalization of Wts from Jub to Ex.

### Wts–Ex binding and Wts activation

**Figure 5:**
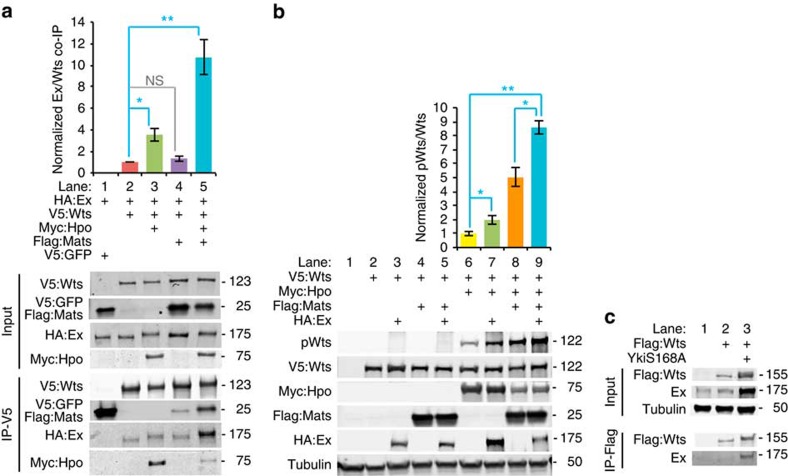
Wts activation complexes. (**a**) Histogram (above) and western blots (below) showing results of co-immunoprecipitation with V5-tagged GFP (lane 1) or V5:Wts (lanes 2–5) on lysates of S2 cells expressing the indicated proteins, and blotted with V5, Flag, Myc or HA antisera. Histogram shows the mean Ex/Wts amounts from four replicates, normalized to the ratio in cells co-expressing only V5:Wts and HA:Ex. Error bars indicate s.e.m., significance determined by *t*-test. (**b**) Histogram (above) and western blots (below) showing activation of Wts (pWts) in S2 cells expressing the indicated proteins. Histogram shows pWts/Wts ratio, from three replicates, normalized to the ratio in cells co-expressing only V5:Wts and Myc:Hpo. Error bars indicate s.e.m., significance determined by *t*-test. (**c**) Western blots showing results of co-immunoprecipitation with Flag-tagged GFP:Wts from lysates of wing discs from wild-type, *GFP:Wts* and *GFP:Wts en-Gal4 UAS-RFP UAS-Yki*^*S168A*^ *tub-Gal80*^*ts*^ shifted to 29 °C for 24 h before dissection. This blot is representative of three independent biological replicates. Numbers to the right of blots indicate molecular weights.

To assess whether the co-localization of Wts and Ex could be reflective of direct association, we assayed for physical interaction between Wts and Ex. Indeed, when tagged forms of Ex and Wts were co-expressed in cultured *Drosophila* S2 cells, we discovered that Ex could be co-precipitated with Wts, but not with a control protein (GFP), indicating that Wts and Ex can interact (Fig. 5a). This co-precipitation was increased approximately threefold when Hpo was co-transfected together with Ex and Wts, and was increased >10-fold when Hpo and Mats were both co-transfected with Ex and Wts (Fig. 5a). Thus, physical association between Wts and Ex is promoted by Hpo and Mats. To confirm that endogenous Wts and Ex can associate *in vivo*, we prepared lysates of wing discs expressing GFP:Wts (which is also Flag tagged). Significant co-precipitation was observed from lysates prepared from discs expressing Yki^S168A^, but not from wild-type wing disc lysates (Fig. 5c). Together with the genetic requirements for Ex, Mats, Sav and Hpo in Wts relocalization, these observations indicate that relocalization of Wts is associated with Wts–Ex binding, and further imply that increased binding between Wts and Ex induced by Hpo and Mats contributes to the relocalization of Wts observed *in vivo* in Yki^S168A^-expressing cells. Intriguingly, we observed a slight shift in Ex mobility when Hpo was co-transfected (Fig. 5a,b), suggesting that Hpo can phosphorylate Ex.

The correlation between Wts relocalization to Ex and Wts activation prompted us to further examine the role of Ex in promoting Wts activation. Detection of Wts activation in S2 cells by western blotting with pWts antisera required co-transfection of Hpo and Wts (Fig. 5b). Ex then further stimulates Wts activation, because addition of Ex increased pWts, both in the absence or presence of exogenous Mats (Fig. 5b). This stimulation of Wts activation could be explained by the ability of Ex to bind to both Hpo^10^ and Wts (Fig. 5a), thereby acting as a scaffold to link them together.

### Wts or Ex overexpression induces pWts *in vivo*

**Figure 6:**
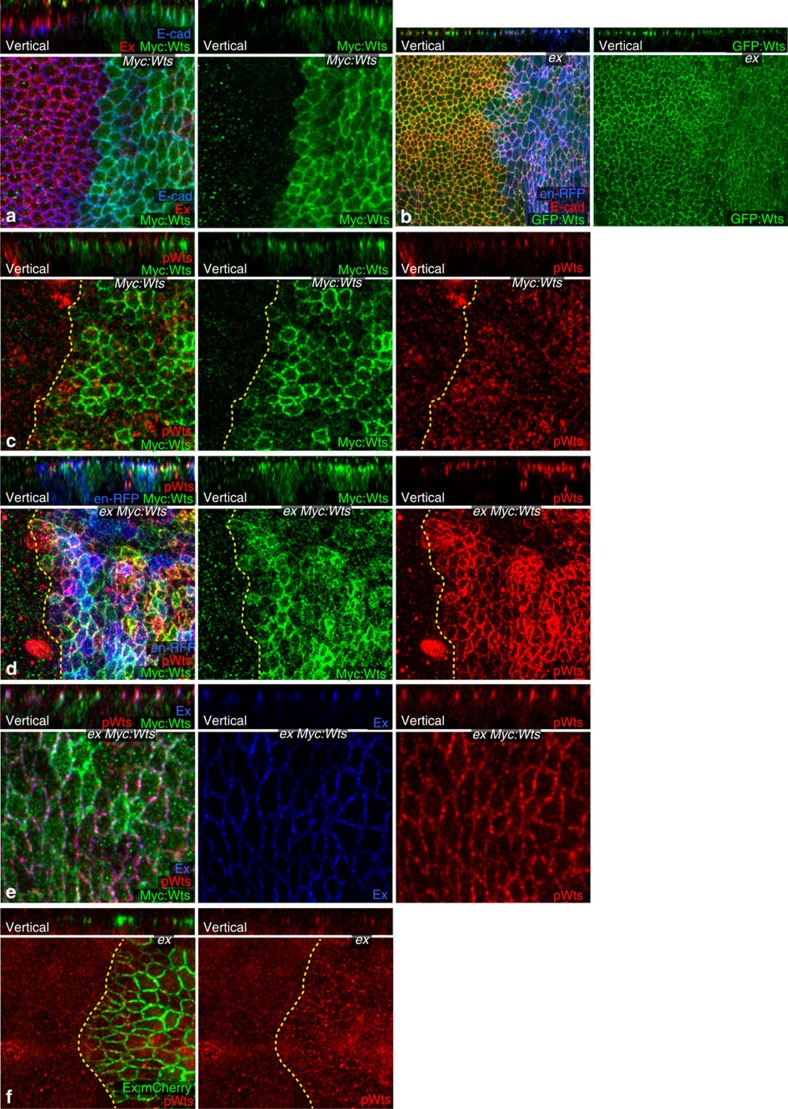
Activation of Wts by Wts or Ex expression. Wing discs transiently expressing transgenes in posterior cells using *en-Gal4 tub-Gal80*^*ts*^ and in some cases *UAS-Dcr2* and/or *UAS-RFP* (blue), including combined and individual stains as indicated, in horizontal and vertical (as marked) sections, dashed yellow line marks A–P boundary. (**a**) Expresses UAS-Myc:Wts (green), stained for E-cad (blue) and Ex (red). (**b**) Expressing GFP:Wts and *UAS-ex*, 24 h under *en-Gal4* control, stained for E-cad (red). (**c**) Wing disc expressing Myc:Wts (green) in posterior cells for 24 h under *en-Gal4* control, and stained for pWts (red). (**d**,**e**) Wing discs expressing UAS-Myc:Wts (green) and UAS-ex, stained for pWts (red), and in **e**, Ex (blue). (**f**) Wing disc expressing *UAS-ex:mCherry* (green) in posterior under control of *en-Gal4 tub-Gal80*^*ts*^, after a 12 h shift to 29 °C, stained for pWts (red).

Expression of Yki^S168A^ could potentially upregulate multiple Hippo pathway components that contribute to relocalization and phosphorylation of Wts *in vivo*. Since activation of Yki increased Wts levels, and overexpression of Wts is sufficient to increase Hippo pathway activity, we investigated the influence of Wts overexpression on its localization and phosphorylation. When Wts was transiently overexpressed using a *UAS-Myc:Wts* transgene under the control of *en-Gal4* and *tub-Gal80*^*ts*^, it could be detected in the cytoplasm, but a distinct sub-apical accumulation was visible, where it overlaps both E-cad and Ex (Fig. 6a). Quantitation revealed similar increases in Wts induced by UAS-Yki^S168A^ and *UAS-Myc:Wts* (Supplementary Fig. 2e). Weak pWts staining was detected overlapping this accumulation of Myc:Wts at apical junctions (Fig. 6c); hence, simply increasing Wts levels is sufficient to enable detection of pWts *in vivo*.

Recently, it was reported that myristoylation of Wts enhanced its activation, presumably through membrane targeting^15^. Interestingly, when expressed in wing disc cells, Myr:V5:Wts was not evenly distributed on membranes as expected for myristoylated proteins, but accumulated at higher levels at apical junctions, overlapping E-cad and Ex (Supplementary Fig. 5a). In contrast, a different myristoylated protein, Myr:Mats:GFP^27^, was evenly distributed on cellular membranes (Supplementary Fig. 5e). These observations suggest that interaction with Jub and Ex exerts a greater influence on Wts localization than the myristoylation tag. Expression of Myr:V5:Wts was also sufficient to induce pWts staining, and pWts accumulation above background was only observed at apical junctions, rather than along all membranes (Supplementary Fig. 5c). As an additional control for pWts staining specificity, we took advantage of a construct expressing a myristoylated Wts protein with a mutation at the Hpo phosphorylation site (Myr:V5:Wts^T1077A^)^15^; this protein accumulated in a similar pattern as wild-type Myr:V5:Wts, but no specific pWts staining was detected (Supplementary Fig. 5b,d). These overexpression experiments with wild-type or myristoylated Wts imply that visible Wts activation only happens at a discrete junctional site where Wts overlaps with Wts activators. Indeed, co-expression of Ex with Wts resulted in a more robust activation of Wts than overexpression of Wts alone (Fig. 6c–e). Quantitation revealed that the increase in Ex by UAS-Yki^S168A^ was two- to threefold greater than that induced by *UAS-ex* (Supplementary Fig. 2e).

To investigate whether other means of activating the Hippo pathway also could be sufficient for detection of Wts relocalization or phosphorylation, we transiently expressed Ex, Hpo, Sav or Myr:Mats for 24 h, using *en-Gal4* and *Gal80*^*ts*^. These reduced total levels of Wts (Fig. 6b; Supplementary Fig. 5f–h), consistent with the discovery that Hippo signalling regulates Wts levels. Some detectable Wts remained at adherens junctions, but this Wts overlaps E-cad rather than Ex, and pWts staining was not detectable. Possibly, the reduced levels of Wts prevented us from detecting pWts. Alternatively, as all signal transduction pathways require mechanisms to reverse pathway activation, the lack of detectable pWts staining without Wts overexpression might reflect a rapid turnover of pWts (for example, via protein degradation or dephosphorylation), such that pWts normally cannot accumulate to high enough levels for detection using available antisera. Conversely when Wts is overexpressed (by direct overexpression or by Yki activation), the machinery dedicated to turnover of pWts might become saturated, allowing pWts to accumulate to detectable levels. Thus, we reasoned that it might be possible to transiently identify pWts production within a short time window after Hippo pathway activation. Indeed, we found that a faint pWts signal could be detected 12 h after induction of Ex expression (Fig. 6f). Thus, pWts can be detected *in vivo* at Ex sites either after increasing Wts or increasing Ex, and a robust pWts signal is visible when both Wts and Ex are increased, either by co-expression or as a consequence of transcriptional upregulation by Yki.

## Discussion

Wts is a key control point within the Hippo pathway, where multiple upstream regulatory processes converge^1^,^2^,^3^. A fundamental gap in our understanding of Hippo signal transduction has been the cellular location of Wts activation. Our observations here establish that Wts activation in wing disc epithelial cells occurs at sub-apical junctions where Hpo, Sav, Ex and Wts overlap. They further implicate co-recruitment of Hpo and Wts kinases to a common scaffold as a central feature of Hippo pathway activation, and help to explain why genes required for apical junctions and apical–basal polarity promote Hippo signalling and can act as tumour suppressors^4^.

**Figure 7:**
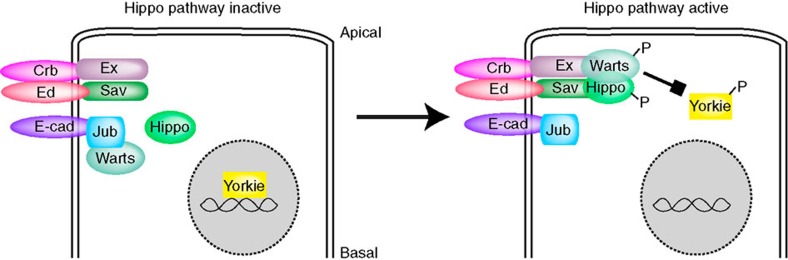
Wts activation is coupled to relocalization of Wts and Hpo. Schematic highlighting relative localization of Hippo pathway components, and the relocalization of Hpo and Wts associated with Wts activation.

Our studies indicate that a key step in Wts activation in disc epithelia is its relocalization from Jub to Ex (Fig. 7). No special mechanism is needed to transport Wts from Jub to Ex, as Wts localization could simply be governed by equilibrium binding with a limited cytoplasmic pool. That is, if Wts normally binds relatively strongly to Jub, and relatively weakly to Ex, it could, depending on its concentration, accumulate at Jub sites but not at Ex sites. Expression of activated Yki induced a robust relocalization of Wts from Jub to Ex, and our studies identify three factors that contribute to the visible accumulation of Wts at Ex sites under these conditions. First (as discussed below), Yki activation appears to increase Hpo activity. We also found that *hpo* RNAi suppressed the relocalization of Wts from Jub to Ex, and that increased Hpo activity promotes Ex–Wts binding, as assayed by co-immunoprecipitation experiments. These observations are consistent with the hypothesis that Wts shifts from Jub sites towards Ex sites due to an increased Ex–Wts binding affinity induced by Hpo. Second, Yki activation increases levels of Ex, which under equilibrium binding would also increase the recruitment of Wts to Ex sites. The relocalization of Wts back to adherens junctions in the absence of Ex indicates that the shift in Wts localization is Ex dependent, and implies that Jub and Ex can compete for association with Wts. A third factor that contributes to detection of Wts–Ex co-localization is the increase in Wts protein levels induced by activated Yki, which could lead to Wts concentrations high enough to bind even lower-affinity Ex sites, and indeed we observed that simply overexpressing Wts was sufficient to induce Wts–Ex overlap, without removing Wts from adherens junctions where it co-localizes with Jub. We suggest that an additional consequence of increased Wts levels that enables detection of Wts and pWts overlapping Ex could be a saturation of pWts removal. While at present this remains speculative, all signal transduction pathways require mechanisms to turn off after they have been activated, so there should exist mechanisms that either degrade or dephosphorylate pWts. Relatively low levels of pWts due to rapid turnover could also help explain why pWts was undetectable in wild-type wing discs.

Our discovery of Ex–Wts binding, together with earlier studies that identified Ex–Hpo binding^10^, implicate Ex as a scaffold that could promote Wts activation by co-localizing it with Hpo, and thus define a role for Ex distinct from previous suggestions that it functions as an activator of Hpo. Similarly, recent studies in cultured cell models showed that activated forms of Mer could bind Wts, and suggested a model in which Mer promotes Wts activation by recruiting it to membranes where it could be activated by Hpo^15^. This suggests that in tissues where Mer, rather than Ex, plays key roles in Wts activation, such as glia^28^, Mer, which can also associate with Hpo, through Sav^10^,^28^, could play an analogous role in assembling a Wts activation complex^15^. It is thus noteworthy that the best characterized upstream branches of ‘Hippo' signalling characterized in *Drosophila*: Fat, Ex and Mer can all now be said to act principally at the levels of Wts regulation rather than Hpo regulation. Moreover, we note that Kibra, which has been suggested to act at a similar point in the Hippo pathway as Mer and Ex^10^,^11^,^12^, has also been reported to be able to physically interact with both Hpo and Wts, and thus might also act principally as a scaffold that links them together rather than as a promoter of Hpo activation.

Indeed, external signals that impinge directly on Hpo activity have not yet been identified. Our discovery that Hpo localization to Sav is greatly increased by Yki activation reveals that regulators of Hpo localization exist, and implies that they are subject to negative feedback regulation downstream of Yki. As Hpo kinase activity can be promoted by Hpo dimerization^29^,^30^, we propose that the increased recruitment of Hpo to Sav could elevate Hpo activity by increasing its local concentration, and thereby its dimerization. Relocalization of Hpo might also affect its interactions with kinases that can modulate Hpo activity^31^,^32^,^33^. Recruitment of Hpo to Sav also concentrates Hpo near Ex, where it would more efficiently phosphorylate Ex-bound Wts. However, since most junctional Wts in disc epithelia is normally complexed with Jub rather than Ex, a mechanism-based solely on Hpo recruitment to apical junctions would not be expected to induce robust Wts activation. Importantly, then, our studies revealed that Hpo can increase Ex–Wts binding, possibly by phosphorylating Ex. Increased Ex–Wts binding would help recruit Wts to Ex, where it could then be phosphorylated by Hpo. Thus, it is now possible to suggest a sequential model for Hippo pathway activation in which Hpo is first recruited to membranes and activated, activated Hpo then phosphorylates Ex to recruit Wts and finally Hpo phosphorylates and activates Wts complexed with Ex. While further studies will be required to validate this model, it provides a framework that could guide future investigations, and our current studies clearly emphasize the importance of determining the *in vivo* localization of endogenous pathway components.

## Methods

### *Drosophila* genetics

Previously described tagged genomic transgenes were used to analyse localization of Crb, Wts or Jub expressed at endogenous levels^18^,^34^,^35^. Overexpression and RNAi experiments employed *en-Gal4* and *tub-Gal80*^*ts*^ (Bloomington stocks (BL) 7108 and 7017) crossed with *UAS-Dcr2* (ref. 36) and *UAS-yki:V5*^*S168A*^ or *UAS-yki:V5* (ref. 26), *UAS-ex*^37^, UAS-ex:mCherry (Y. Feng, 2009), *UAS-sav* and *UAS-hpo*^38^, *UAS-myr:V5:Wts* and *UAS-myr:V5:Wts*^*T1077A*^ (ref. 15), *UAS-Myc:Wts*^39^ *UAS-Myr:Mats:GFP*^27^, *UAS-jub-RNAi* (vdrcGD38442), *UAS-ex-RNAi* (BL34968), *UAS-sav-RNAi* (vdrcKK101323), *UAS-hpo-RNAi* (vdrcKK104169), *UAS-mats-RNAi* (vdrcKK108080), *UAS-wts-RNAi* (vdrcGD9928) and *UAS-Mer-RNAi* (vdrcGD7161), with temperature shifts to 29 °C for 16–24 h before dissection. The effectiveness of *hpo*, *mats*, *Mer* and *sav* RNAi is shown in Fig. 4b, Supplementary Figs 1c,d and 3d, other RNAi lines have been validated in earlier publications.

A genomic construct expressing GFP:Mats was created by inserting the GFP tag, including splice acceptor and donor sequences, from a MiMIC construct^40^ into the first coding intron of a genomic *mats* construct, which was created by PCR using primers: MatsupF, 5′- TTTCCCCTTGCTGTACAAAGTT -3′; MatsupR, 5′- AAGCTATTTTTTAAACACCGCAGA -3′; MatsdownF 5′- TTCAAAGATATCTTGCATTCTTCCTC -3′; MatsdowR, 5′- GATTTGCCTATGCGAAATGCCG -3′, which encompass 3-kb upstream and 3-kb downstream of this intron. After confirmation by DNA sequencing, the construct was injected and integrated into the attP2 site.

### Immunofluorescence and image quantitation

Dissected wing discs were fixed in 4% paraformaldehyde for 8 min and stained using as primary antibodies rat anti-Ed (1:50, J.-C. Hsu), rabbit anti-Sav (1:200, J. Jiang), rat anti-E-cad (1:100, DSHB DCAD2), guinea pig anti-Ex (1:2,000, R. Fehon), guinea pig anti-Hpo (1:200, G. Halder), anti-Wts, rabbit anti-pWts (1:50, D. Pan) and mouse anti-V5 (1:100, Invitrogen, R960-25), mouse anti-ß-gal (1:400, DSHB JIE7-c), mouse anti-Myc (1:100, Covance MMS-150R); and as secondary antibodies goat anti-mouse-555 (3:400, Invitrogen, A21424), goat anti-mouse-488 (3:400, Invitrogen, A11029), goat anti-guinea pig-555 (3:400, Invitrogen, A21435), goat anti-guinea pig-633 (1:100, Invitrogen, A21105), goat anti-rabbit-555 (3:400, Invitrogen, A27039), goat anti-rabbit-633 (1:100, Invitrogen, A21072), donkey anti-rat-647 (1:100, Jackson, 712-605-150) and donkey anti-rat-Cy3 (3:400, Jackson, 712-165-150). Confocal images were captured on a Leica SP5. Pearson's correlation coefficient^41^, and the distance between centroids of Ex and Wts puncta, was calculated using Volocity software (PerkinElmer). Horizontal images shown are projections through 10–20 sections, encompassing a slice of at least 2 μm, and include all of the apical junctions studied. The optical resolution of the × 63 1.4 numerical aperture objective used is estimated to be ∼0.4–0.5 μm in the *z* axis with a 1-Airy unit pinhole, and hence sufficient to detect the estimated separation of 0.8 μm. Statistical analysis (*t*-tests for pairwise comparisons) was performed using Graphpad Prism software.

### Cell culture, immunoblotting and immunoprecipitation

S2 cells were transfected with plasmids pAC5.1-Wts:V5 (D. Pan), pAC5.1-HA:Ex (G. Halder), pAC5.1-Myc:Hpo (D. Pan), pUAST-Flag:Mats (constructed by PCR amplifying Mats and an N-terminal Flag tag into pUAST), aw-Gal4 or pAC5.1-Flag:GFP^28^ using Effectene (Qiagen). Cells were lysed in RIPA buffer with protease inhibitor cocktail (Roche) and phosphatase inhibitor cocktail (Calbiochem). For immunoprecipitation, anti-V5 agarose beads (Sigma) were incubated with cell lysates at 4 °C for 4 h, and then washed four times. Protein samples were run on 4–15% gradient gels (Bio-Rad). Immunoblotting was performed using as primary antibodies rabbit anti-Myc (Santa Cruz, sc-789, 1:5000), mouse anti-Flag M2 (Sigma, F3165, 1:5,000), rabbit anti-HA (Abcam, ab9110, 1:5,000), mouse anti-V5 (Invitrogen, P/N46–0705, 1:5,000), guinea pig anti-Ex (R. Fehon, 1:5,000) and rabbit anti-pWts (1:1,000, D. Pan)^10^, and as secondary antibodies goat anti-mouse 680 (Li-Cor, 926–68,020), goat anti-rabbit-680 (Li-Cor, 926–68,021), goat anti-rabbit 800 (Li-Cor, 926–32,211), donkey anti-guinea-pig-680 (Li-Cor, 926–68,077) and donkey anti-gunea-pig-800 (Li-Cor, 926–32,411), all at a dilution of 1:5,000. Blots were scanned and bands quantified using an Odyssey Imaging System (Li-Cor). Wts and Hpo levels in wing discs were determined by lysing discs in loading buffer (20 discs per lane) from wild type, *GFP:Wts* and *GFP:Wts en-Gal4 tub-Gal80*^*ts*^ *UAS-Yki:V5*^*S168A*^ after shifting to 29 °C for 24 h, and blotting lysates using anti-Hpo (G. Halder, 1:2,000), anti-Flag (GFP:Wts is also Flag tagged, Sigma, F3165, 1:5,000), and for normalization anti-GAPDH (Imgenex, IMG-6665 A-25, 1:10,000) primary antibodies. For wing disc co-immunoprecipitation, 100 discs from wild-type, *GFP:Wts*, and *GFP:Wts en-Gal4 UAS-Yki*^*S168A*^ *tub-Gal80*^*ts*^, shifted to 29 °C for 24 h were lysed and incubated with mouse anti-Flag beads (Sigma, Sigma, A2220; the tag in GFP Wts includes a Flag epitope tag), and analysed by western blotting using guinea pig anti-Ex (R. Fehon, 1:5,000), mouse anti-α-tub (Sigma, T6199, 1:10,000) or mouse anti-Flag (Sigma, F3165, 1:5,000). Three independent biological replicates were performed; all gave similar results. Uncropped blots are shown in Supplementary Fig. 6.

## Additional information

**How to cite this article:** Sun, S. *et al*. Localization of Hippo signalling complexes and Warts activation *in vivo*. *Nat. Commun.* 6:8402 doi: 10.1038/ncomms9402 (2015).

## Supplementary Material

Supplementary Information Supplementary Figures 1-6
