# Supplementary material for: Localization of Hippo signalling complexes and Warts activation in vivo
Source: Nat Commun. 2015 Sep 30;6:8402. doi: 10.1038/ncomms9402 (PMC4598633; doi:10.1038/ncomms9402)

Supplementary Information

Supplementary Figures

Supplementary Figure 1 Apical organization of Hippo pathway components

a) Wing disc cells expressing Crb:GFP (green) stained for Sav (red) and E-cad (blue), including combined and individual stains as indicated, in horizontal and vertical (as marked) sections, yellow arrows highlight Crb:GFP, white arrow highlights E-cad, yellow scale bars indicate 3µm. b) Wing disc cells stained for E-cad (green) and Hpo (red), including combined and individual stains as indicated, in horizontal and vertical (as marked) sections, yellow scale bar indicates 3µm. c) Wing disc expressing *en-Gal4 UAS-GFP UAS-dcr2* and *UAS-hpo-RNAi*, stained for Hpo (red) to confirm antisera specificity, including combined and individual stains as indicated, in horizontal and vertical (as marked) sections. d) Wing disc expressing *en-Gal4 UAS-dcr2 Mer:GFP* and *UAS-Mer-RNAi*, to confirm effectiveness of *Mer* RNAi, including combined and individual stains as indicated, in horizontal and vertical (as marked) sections. Mer:GFP was created within a BAC clone and will be described elsewhere.

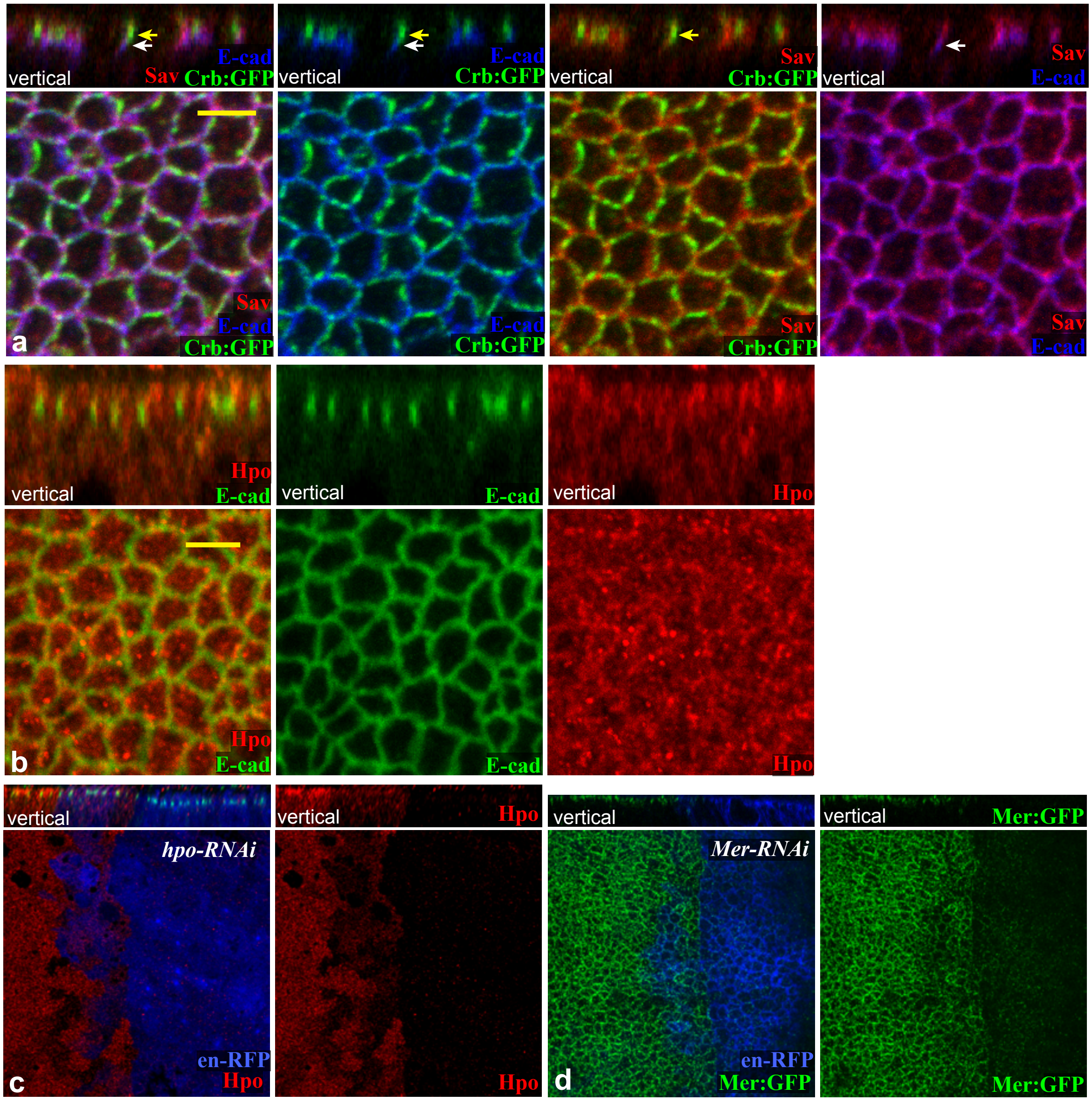
vertical
E-cad
Sav
Crb:GFP
a
b
c
d
Hpo
hpo-RNAi
en-RFP
Mer-RNAi
Mer:GFP

Supplementary Figure 2 Wts regulation and pWts antisera specificity

a) Wing disc expressing GFP:Wts (green) and *en-Gal4 UAS-RFP (blue) UAS-dcr2 UAS-wts-RNAi* stained for pWts (red). All of the signal with pWts anti-sera here is presumed to be background, because it is unaffected by Wts RNAi. b) Wing discs with *wts-LacZ* transgene (wts^{P2}) expressing *UAS-Yki*^{S168A} in dorsal cells under *ap-Gal4 tub-Gal80*^{ts} control, after shifting to 29°C for 24h, marked by *UAS-RFP* (blue), and stained for ß-gal (green). c) Wing disc expressing GFP:Wts and *en-Gal4 UAS-RFP* (blue) *UAS-wts-RNAi* , after shifting to 29°C for 24h, and stained for pWts (red). d) Wing disc expressing GFP:Wts (green) *en-Gal4 UAS-Yki:V5*, stained for V5 (red). All panels include combined and individual stains as indicated, in horizontal and vertical (as marked) sections. e) Representative western blots on the indicated proteins (molecular weight on right) and results of quantification of three independent blots performed on wing discs lysates from *en-Gal4 tub-Gal80*^{ts} and *UAS-yki:V5*^{S168A} *, UAS-myc:wts, UAS-ex, or UAS-RFP (control)* shifted to *29*°C 24h before dissection; amounts were quantified and normalized to Tubulin, and represented as the mean amount of Wts or Ex protein in each genotype relative to that in control animals. Error bars indicate s.d.

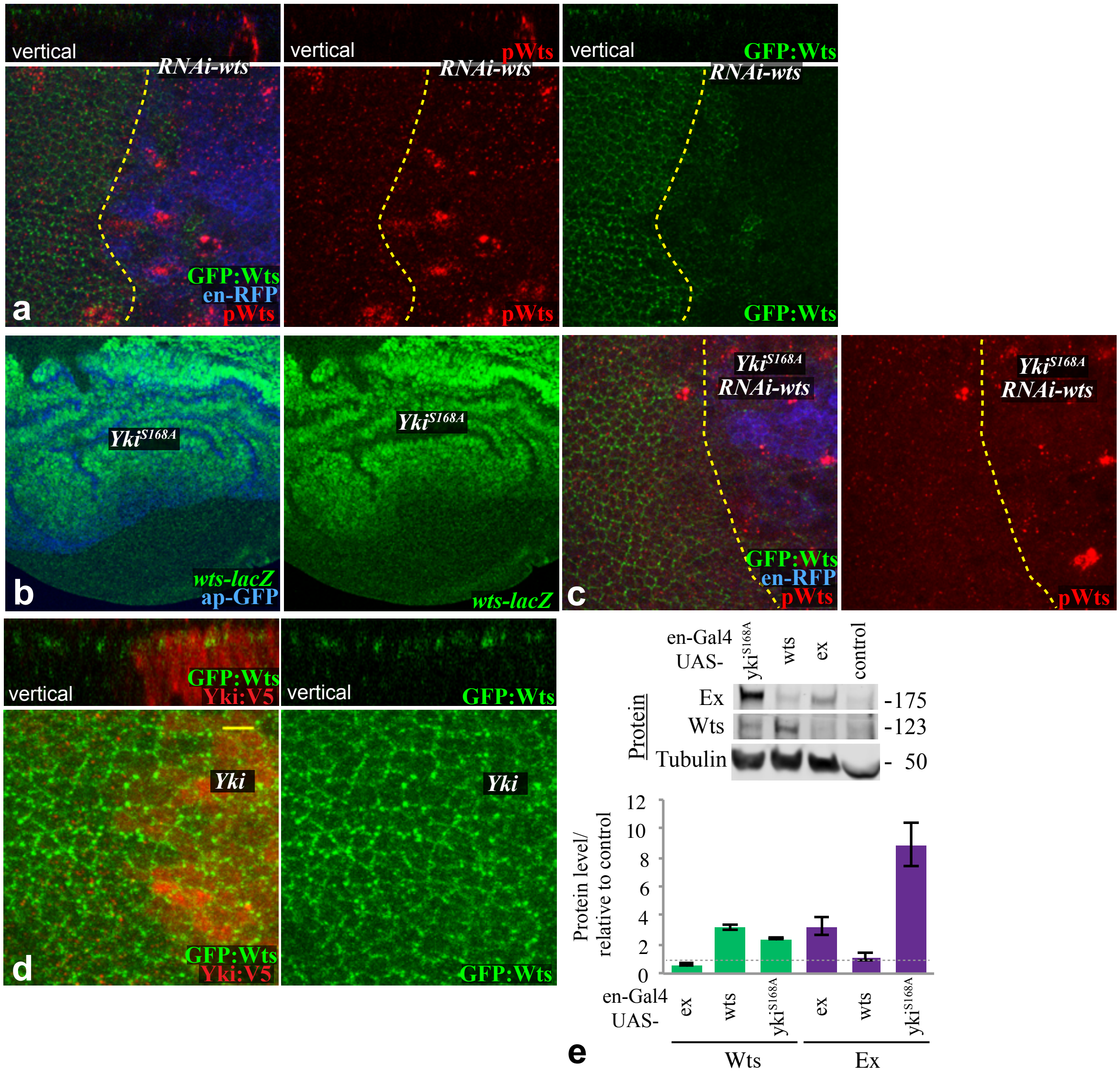
vertical
RNAi-wts
pWts
GFP:Wts
en-RFP
a
b
YkiS168A
wts-lacZ
ap-GFP
c
YkiS168A RNAi-wts
d
Yki
Yki:V5
en-Gal4
UAS-
ykiS168A
wts
ex
control
Protein
Ex
Wts
Tubulin
-175
-123
- 50
Protein level/ relative to control
12
10
8
6
4
2
0
e

Supplementary Figure 3 Wts activation and relocalization

a) Eye disc expressing GFP:Wts with clones (marked by *UAS-2xEBFP*, blue) expressing *UAS-Yki*^{S168A} under control of an Actin>y+>Gal4 Flip-out cassette*,* and stained for E-cad (red) to mark adherens junction**.** b) Wing disc expressing Jub:GFP, and in posterior cells, Yki:V5^{S168A} for 24h under *en-Gal4* control, marked by *UAS-RFP* (blue), and stained for pWts (red). Panels marked by "–v" show vertical sections, panels marked by numbers show higher magnification of the boxed region. c) Wing disc expressing GFP:Wts and in posterior cells marked by *UAS-RFP* (blue) for 24h under *en-Gal4* control, Yki:V5^{S168A} and *jub* RNAi, stained for E-cad (red). Panels marked by prime symbols show subsets of channels, panels marked by "–v" show vertical sections, panels marked by numbers show higher magnification of the boxed regions (-1 for anterior cells, -2 for posterior cells). d) Wing disc expressing *sav* RNAi in posterior cells for 24h under *en-Gal4* control, marked by *UAS-RFP* (blue), and stained for Sav (green) and Hpo (red). Panels marked by numbers show higher magnification of the boxed regions (-1 for anterior cells, -2 for posterior cells); although sav RNAi is only partially effective, it nonetheless reduces apical Hpo. e) Wing disc expressing GFP:Wts and in posterior cells 24h under *en-Gal4* control, Yki:V5^{S168A} and *ex* RNAi, stained for Ex (blue) and pWts (red). f) Wing disc expressing GFP:Wts and in posterior cells 24h under *en-Gal4* control, Yki:V5^{S168A} and *sav* RNAi, stained for Ex (blue) and pWts (red). All panels include combined and individual stains as indicated, in horizontal and vertical (as marked) sections.

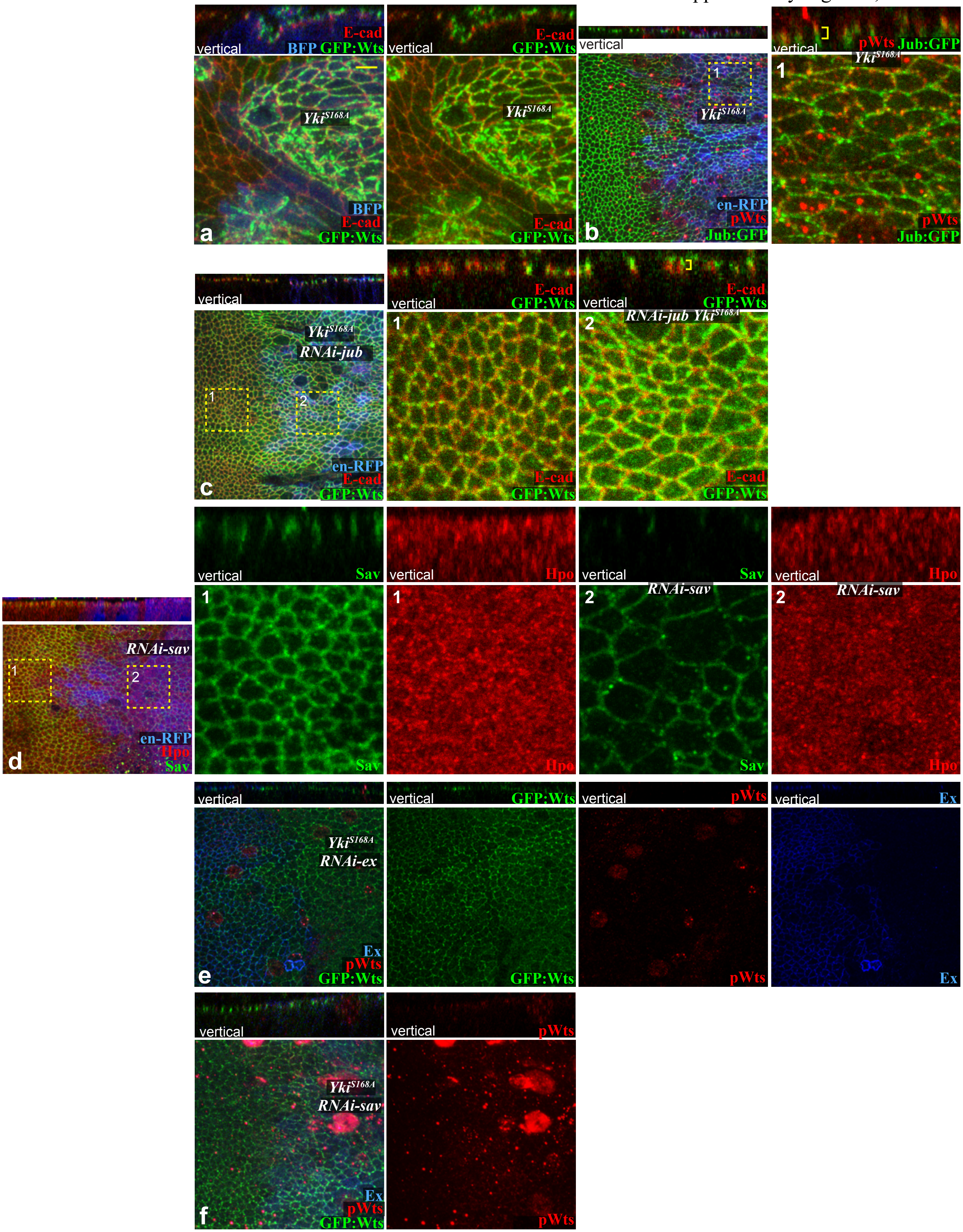
vertical
E-cad
BFP GFP:Wts
YkiS168A
BFP
E-cad
GFP:Wts
a
vertical
E-cad
GFP:Wts
YkiS168A
E-cad
GFP:Wts
vertical
1
YkiS168A
en-RFP
pWts
Jub:GFP
b
vertical
pWts Jub:GFP
YkiS168A
1
pWts
Jub:GFP
vertical
YkiS168A
RNAi-jub
1
2
en-RFP
E-cad
GFP:Wts
c
vertical
E-cad
GFP:Wts
1
E-cad
GFP:Wts
vertical
E-cad
GFP:Wts
RNAi-jub YkiS168A
2
E-cad
GFP:Wts
RNAi-sav
1
2
en-RFP
Hpo
Sav
d
vertical
Sav
1
Sav
vertical
Hpo
1
Hpo
vertical
Sav
RNAi-sav
2
Sav
vertical
Hpo
RNAi-sav
2
Hpo
vertical
YkiS168A
RNAi-ex
Ex
pWts
GFP:Wts
e
vertical
GFP:Wts
GFP:Wts
vertical
pWts
pWts
vertical
Ex
Ex
vertical
YkiS168A
RNAi-sav
Ex
pWts
GFP:Wts
f
vertical
pWts
pWts

Supplementary Figure 4 Genetic requirements for Wts re-localization

Wing discs expressing GFP:Wts, and, in posterior cells for 24h under *en-Gal4* control, marked by *UAS-RFP* (blue), Yki:V5^{S168A} and the indicated RNAi lines. Panels include combined and individual stains as indicated, in horizontal and vertical (as marked) sections, panels marked by numbers show higher magnification of the boxed regions (-1 for anterior cells, -2 for posterior cells): a) Also expresses *UAS-RNAi-Mer*, and stained for E-cad (red). b) Also expresses *UAS-RNAi-hpo*, and stained for E-cad (red). c) Also expresses *UAS-RNAi-hpo*, and stained for Ex (red). d) Also expresses *UAS-RNAi-mats*, and stained for E-cad (blue) and Ex (red). e) Also expresses *UAS-RNAi-sav*, and stained for E-cad (red). f) Also expresses *UAS-RNAi-sav*, and stained for Ex (magenta). For e,f only high magnification images are shown, from anterior or posterior (as marked) regions of the same wing disc. Quantitation of co-localization is in Fig. 2f,g.

Supplementary Figure 4,
Sun et al

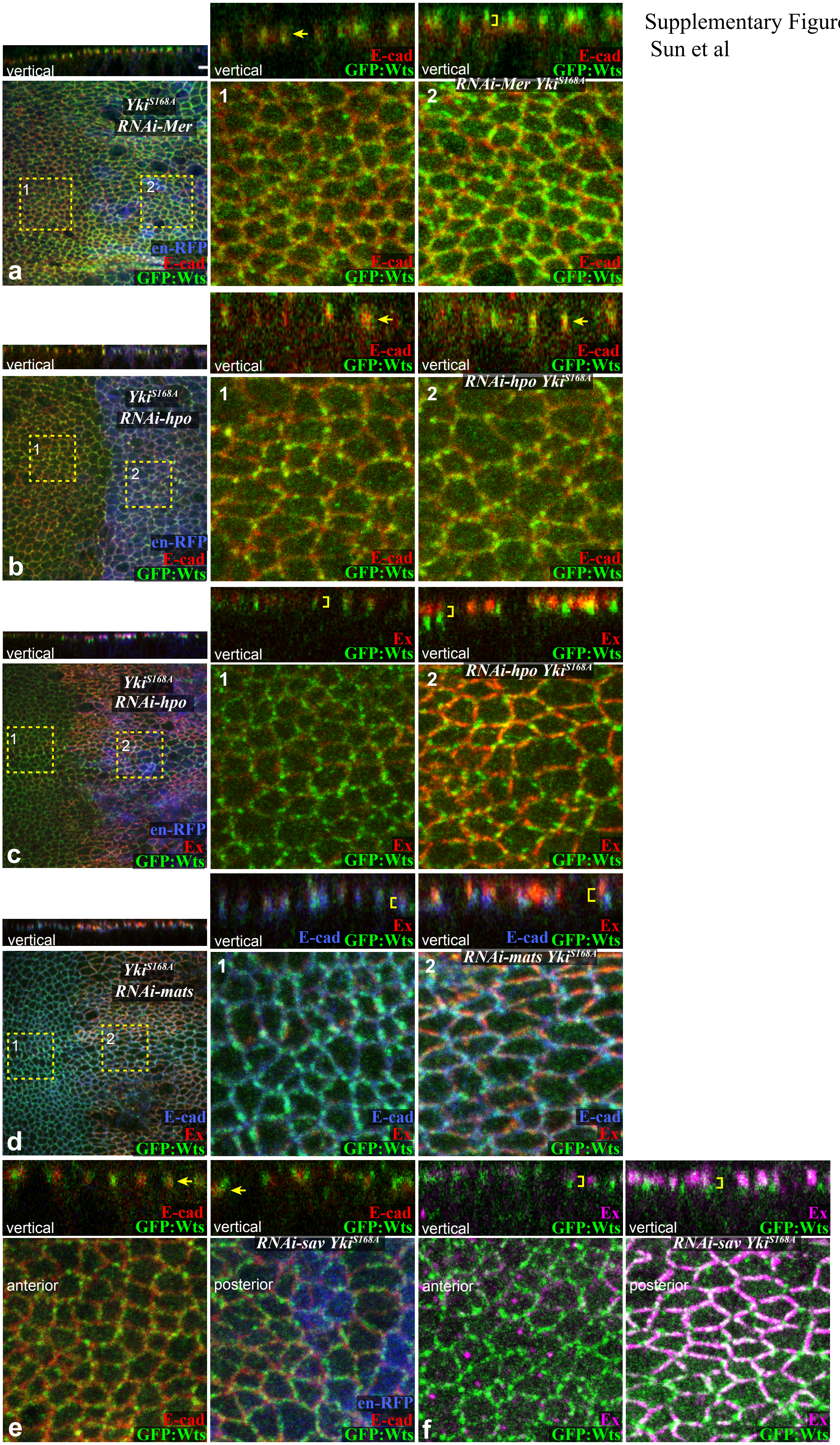

Supplementary Figure 5 Influence of Wts expression on Wts activity and localization

Wing discs transiently expressing transgenes in posterior cells using *en-Gal4 tub-Gal80*^{ts} and in some cases *UAS-Dcr2* and/or *UAS-RFP* (blue). Panels include combined and individual stains as indicated, in horizontal and vertical (as marked) sections. a) Expresses UAS-Myr:V5:Wts (green), stained for E-cad (blue) and Ex (red). b,d) Express UAS-Myr:V5:Wts^{T1077A} (green), stained for pWts or Ex (red). c) Posterior wing disc cells expressing Myr:V5:Wts (green) for 24h under *en-Gal4* control, and stained for pWts (red). Yellow scale bars indicate 5µm. e) Expressing UAS-Myr:Mats (green), stained for pWts (red). f-g) Expressing GFP:Wts and *UAS-hpo* (f) or -*sav* (g), stained for E-cad (red). h) Wing disc expressing GFP:Wts and in posterior cells Hpo for 24h under *en-Gal4* control, marked by *UAS-RFP* (blue), and stained for pWts (red).

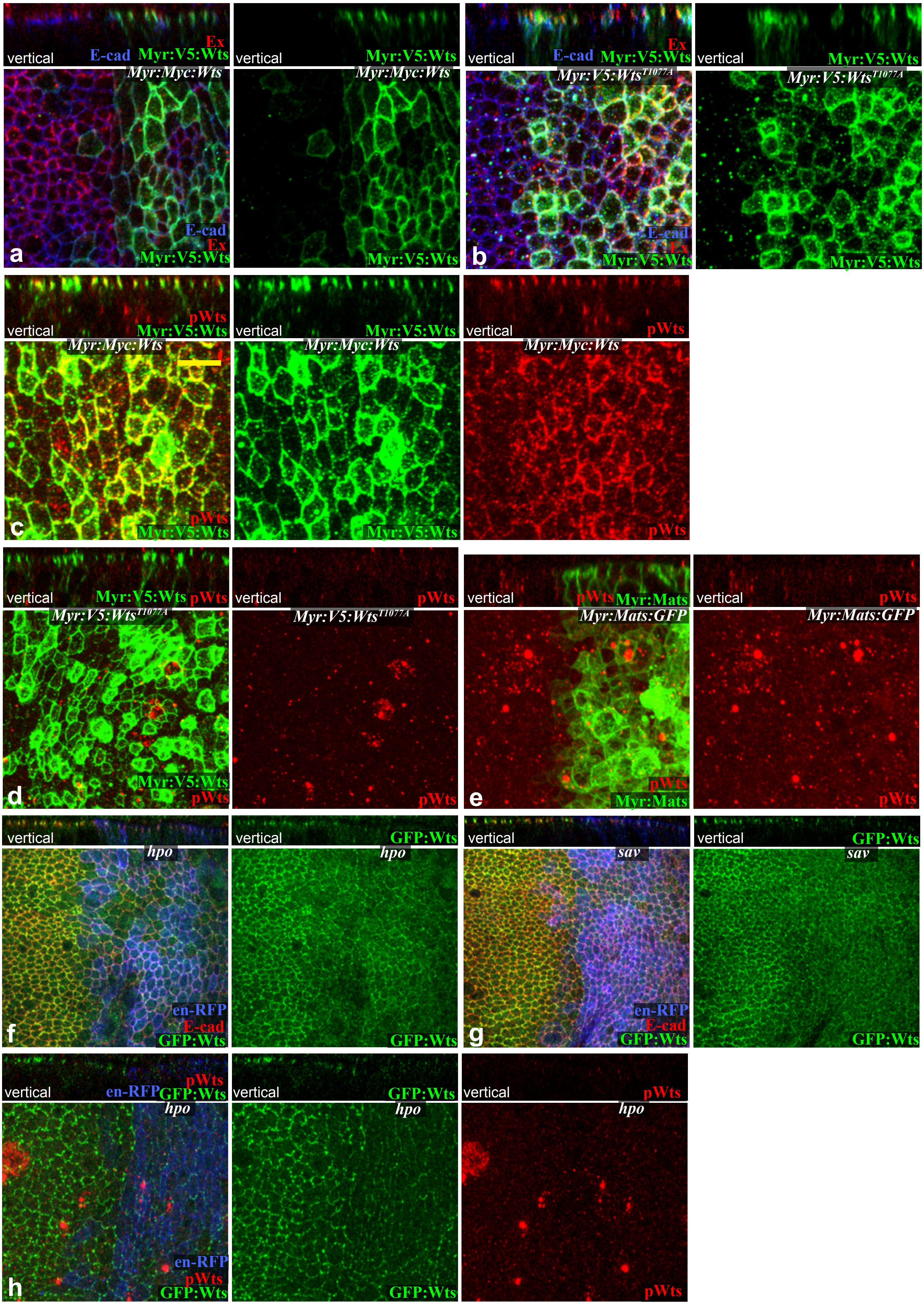
vertical
E-cad
Myr:V5:Wts
Ex
Myr:Myc:Wts
Myr:V5:Wts^T1077A
pWts
Myr:Mats
Myr:Mats:GFP
hpo
sav
GFP:Wts
en-RFP
a
b
c
d
e
f
g
h

Supplementary Figure 6 Uncropped Western blots

Three replicates for each of the bots shown in the figures are shown. The approximate regions shown in the figures are indicated by the red dashed rectangles. Positions of molecular weight markers (in kD) are indicated to the left of each blot. Membranes were cut after transfer so that multiple primary antibodies could be used on the same blot without stripping and re-probing, and to minimize the amount of primary antibodies used. Membranes were stained with the indicated primary antibodies and then secondary antibodies coupled to IRDye 680 and IRDye 800, as indicated. For Fig. 2b, membranes were cut at the 100 kD and 50 kD molecular weight markers. The top third was stained with anti-Flag (for Wts) and anti-Ex, the middle third was stained with anti-Hpo, and the bottom third was stained with anti-GAPDH. For Supplementary Fig. 2e, membranes were cut above 150 and below 100 kD markers. Upper third was stained with anti-Ex, middle third was stained with anti-Wts, bottom third was stained with anti-alpha tubulin. The loading order for repeats 2 and 3 is opposite from that for repeat 1. For 5a, membranes were cut at the 100 kD and 50 kD molecular weight markers. The top third was stained with anti-V5 and anti-HA, the middle third was stained with anti-Myc, and the bottom third was stained with anti-Flag and anti-V5. The two right-most lanes were for an experiment not included in the manuscript. For 5b, membranes were cut at the 100 kD molecular weight marker. The top half was stained with anti-pWts, anti-V5 and anti-HA, the bottom half was stained with anti-Tubulin, anti-Flag and anti-Myc. For Fig 5c, blots were stained for alpha-tubulin, Ex, and Flag.

Blots for Fig. 2b

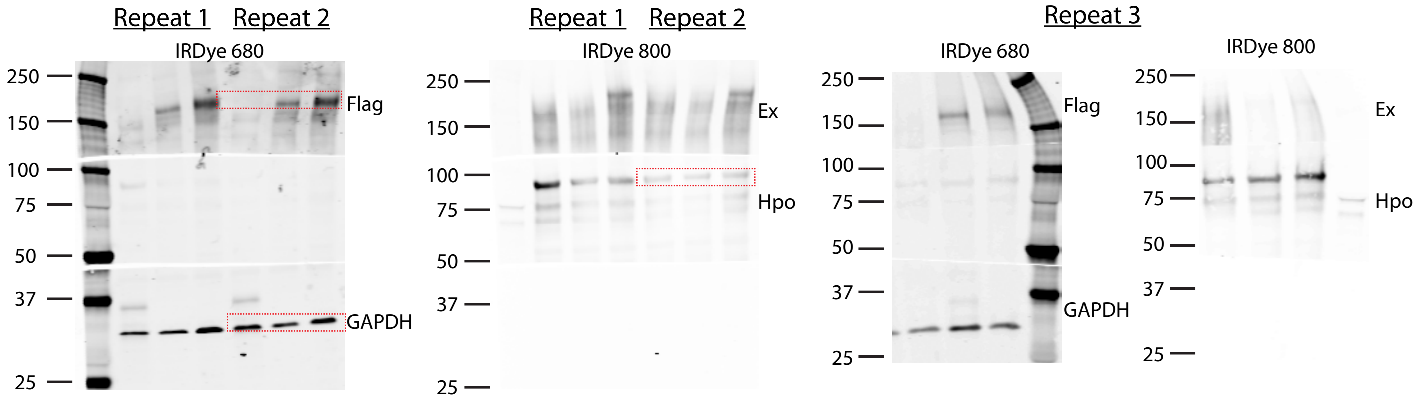

Blots for Fig. S2

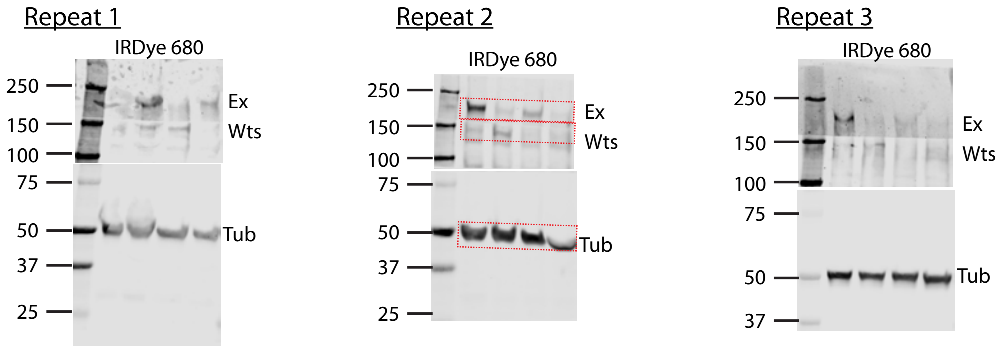

Blots for Fig. 5a

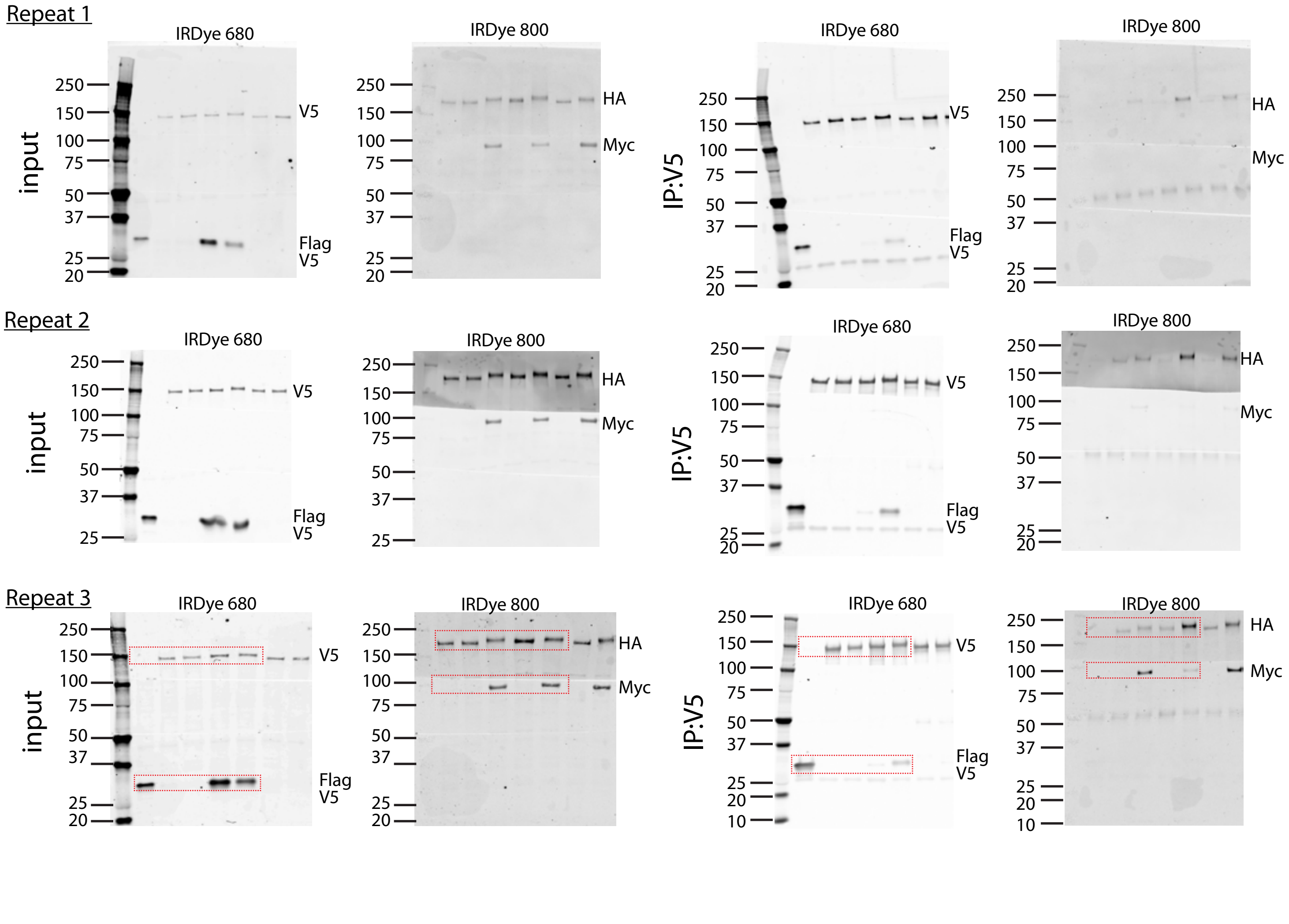

Blots for Fig. 5b

Repeat 1

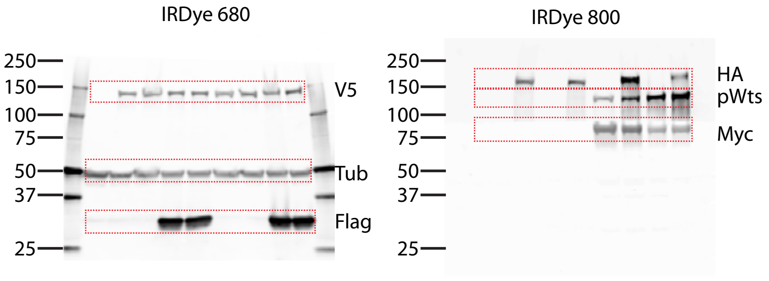

Repeat 2

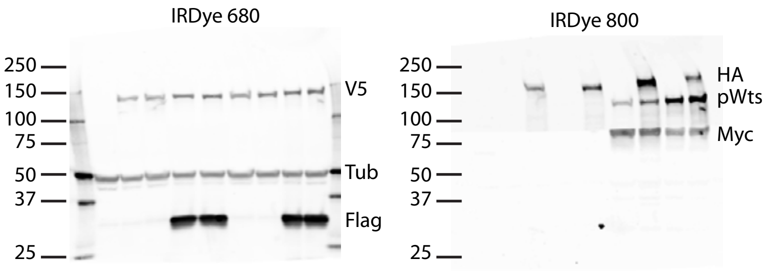

Repeat 3

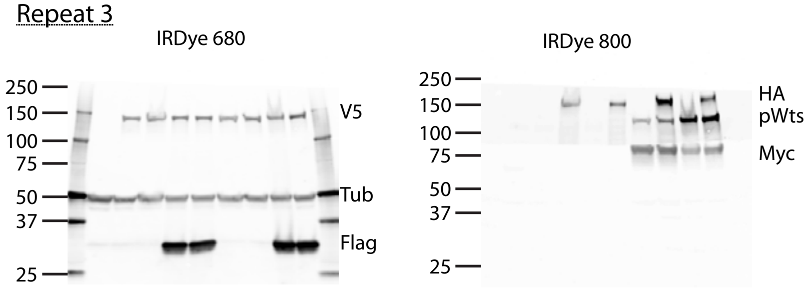

Blots for Fig. 5c

Repeat 1

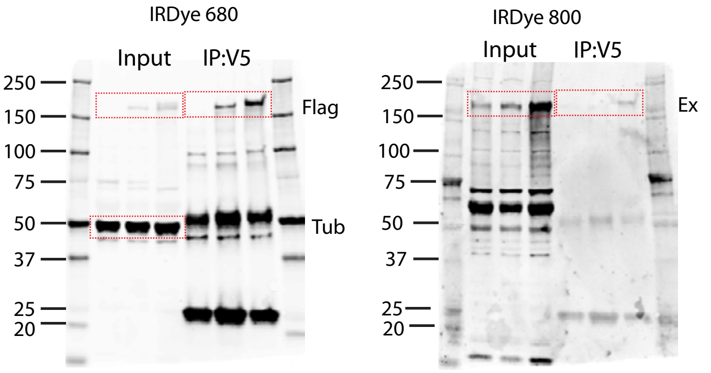

Repeat 2

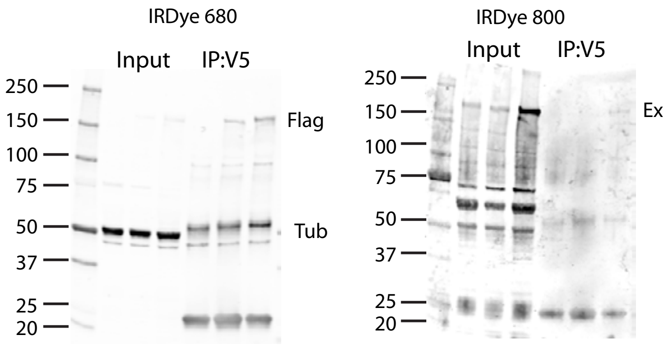

Repeat 3

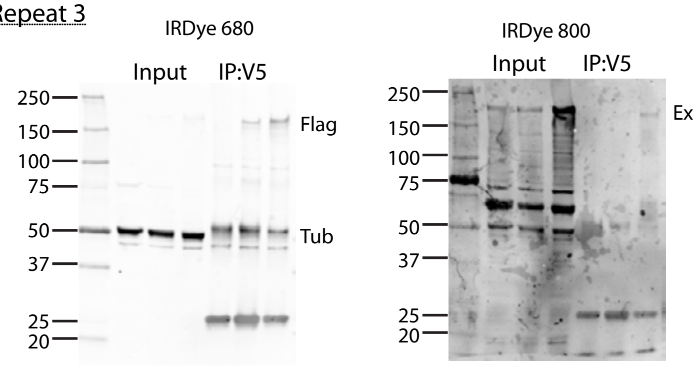

Supplement: Supplementary Information — Supplementary Figures 1-6 [file ncomms9402-s1.pdf]
